# Oxygen‐Doped 2D In_2_Se_3_ Nanosheets with Extended In‐Plane Lattice Strain for Highly Efficient Piezoelectric Energy Harvesting

**DOI:** 10.1002/advs.202410851

**Published:** 2024-11-26

**Authors:** Ji Yeon Kim, Woohyun Hwang, Seo Yeon Han, Ye Seul Jung, Fengyi Pang, Wenhu Shen, Cheolmin Park, Sang‐Woo Kim, Aloysius Soon, Yong Soo Cho

**Affiliations:** ^1^ Department of Materials Science and Engineering Yonsei University Seoul 03722 Republic of Korea; ^2^ SK Hynix Icheon Gyeonggi‐do 17336 Republic of Korea

**Keywords:** 2D materials, α‐In_2_Se_3_, oxygen plasma, piezoelectric energy harvesting, strain engineering

## Abstract

With the emergence of electromechanical devices, considerable efforts have been devoted to improving the piezoelectricity of 2D materials. Herein, an anion‐doping approach is proposed as an effective way to enhance the piezoelectricity of α‐In_2_Se_3_ nanosheets, which has a rare asymmetric structure in both the in‐plane and out‐of‐plane directions. As the O_2_ plasma treatment gradually substitutes selenium with oxygen, it changes the crystal structure, creating a larger lattice distortion and, thus, an extended dipole moment. Prior to the O_2_ treatment, the lattice extension is deliberately maximized in the lateral direction by imposing in situ tensile strain during the exfoliation process for preparing the nanosheets. Combining doping and strain engineering substantially enhances the piezoelectric coefficient and electromechanical energy conversion. As a result, the optimal harvester with a 0.9% in situ strain and 10 min plasma exposure achieves the highest piezoelectric energy harvesting values of ≈13.5 nA and ≈420 µW cm^−2^ under bending operation, outperforming all previously reported 2D materials. Theoretical estimation of the structural changes and polarization with gradual oxygen substitution supports the observed dependence of the electromechanical performance.

## Graphical abstract

Maximizing electromechanical energy conversion in 2D materials requires structural manipulation with extra lattice extension for better power generation performance. Here, a way of extending lattices in a favorable direction using the strain and doping engineering approaches is proposed. Applying two approaches for the optimization of oxygen‐doping level in tensile‐strain‐applied flat α‐In_2_Se_3_ nanosheets produces a remarkable energy‐harvesting result with a record power density of 420 µW cm^−2^.

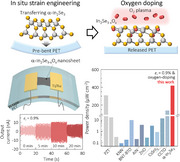

## 1 Introduction

Due to asymmetric crystal structures, numerous 2D materials have been recognized to possess in‐plane or out‐of‐plane piezoelectricity.^[^ ^1^, ^2^, ^3^, ^4^, ^5^ ^]^ Most 2D piezoelectric materials have intrinsic limitations in inducing sufficient polarization depending on the number of layers and the dipole orientation.^[^ ^6^, ^7^, ^8^, ^9^, ^10^ ^]^ For instance, piezoelectricity in 2H‐MoS_2_ exists only in an odd number of layers; otherwise, it causes the electric dipoles to offset each other with the 180° rotation between adjacent layers.^[^ ^6^ ^]^ Specific polarization directions relative to the atomic arrangements can limit the piezoelectricity of 2D materials.^[^ ^7^, ^8^, ^9^, ^10^, ^11^, ^12^ ^]^ Interestingly, strong ferroelectricity has been recently observed in unusual 2D materials having unique crystal anisotropy.^[^ ^13^, ^14^, ^15^ ^]^ As an example, α‐In_2_Se_3_ is known to rarely exhibit strong piezoelectricity in both the in‐plane and out‐of‐plane directions,^[^ ^16^, ^17^ ^]^ which is not common in other 2D materials, which typically possess only lateral (e.g., MoS_2_,^[^ ^6^ ^]^ h‐BN,^[^ ^18^ ^]^ and SnS^[^ ^19^ ^]^) or vertical (e.g., CuInP_2_S_6_,^[^ ^20^ ^]^ WTe_2_,^[^ ^21^ ^]^ and d1T‐MoTe_2_^[^ ^22^ ^]^) electric polarization. Following initial theoretical calculations, α‐In_2_Se_3_ was experimentally investigated to verify the presence of spontaneous polarization in the form of nanosheets ranging from tens of nanometers down to the thickness of the monolayer.^[^ ^23^, ^24^, ^25^ ^]^ α‐In_2_Se_3_ has the rhombohedral *R3m* structure,^[^ ^26^ ^]^ and a single layer of α‐In_2_Se_3_ comprises a quintuple atomic layer consisting of alternating In and Se. The interlinking of in‐plane and out‐of‐plane dipoles is facilitated by a shift in the central Se atom in α‐In_2_Se_3_, which generates a considerable dipole moment.^[^ ^27^, ^28^ ^]^

To facilitate the use of 2D piezoelectric nanomaterials in low‐power nanoscale energy harvesting applications, it is necessary to design and optimize high‐performance materials with enhanced piezoelectric properties. Several methods can modulate the lattice dimension to improve the dipole moment and piezoelectricity, including strain engineering for additional lattice extension and doping engineering for local strain fields, as typically reported in perovskite thin films.^[^ ^29^, ^30^, ^31^, ^32^ ^]^ Recently, a few efforts have been made to engineer the lattice to achieve extended polarization in 2D materials.^[^ ^33^, ^34^, ^35^ ^]^ As the sole example of strain engineering for piezoelectric energy conversion, a wavy structure created in a poly(methyl methacrylate) (PMMA)‐supported MoS_2_ monolayer increased the output voltage six‐fold higher owing to induced strain.^[^ ^33^ ^]^ Regarding doping, the output current of an Au‐doped MoS_2_ monolayer was doubled to ≈100 pA, owing to the reduced screening effect.^[^ ^34^ ^]^ The substitution of Mo with W in monolayer MoS_2_ demonstrated an impressive output current of ≈250 pA, which was attributed to the highest configuration entropy.^[^ ^35^ ^]^

Herein, we introduce a unique strain‐engineering process that has not yet been applied to 2D materials. This method enables intentionally imposing controllable in situ strain in α‐In_2_Se_3_ nanosheets on a polyethylene terephthalate (PET) substrate. The strain engineering process is executed during the wet transfer of the exfoliated nanosheets onto a pre‐bent concave substrate, intended to induce a lateral tensile strain of up to ≈0.9% in the flat state. The level of in situ strain *ε_i_* is controlled through the bending curvature of the substrate.^[^ ^36^, ^37^, ^38^ ^]^ To further modulate the lattice, the structure of α‐In_2_Se_3_ is deliberately modified with oxygen via an O_2_ plasma treatment, resulting in anion substitution in the nanosheets. The doping level of oxygen is carefully controlled by adjusting the plasma conditions, such as exposure time, power, and gas flow rate. O_2_ plasma treatment has been used in some 2D nanosheets, e.g., ReS_2_^[^ ^39^ ^]^ and GeS,^[^ ^40^ ^]^ to introduce vacancies or substituted oxygen to improve charge transfer for other electronic applications. It should be emphasized that the anion‐doping effect, along with the in situ straining method, has never been reported for any piezoelectric energy harvesters based on 2D materials. Particularly, it will be interesting to see how the lattice extension maximized with the combined in situ straining method and doping engineering affects the piezoelectricity and thus the electromechanical power generation.

As a result of the enhanced piezoelectricity with unusual lattice expansion, the optimal piezoelectric energy harvester with α‐In_2_Se_3_ nanosheets reached output levels of ≈13.5 nA and ≈420 µW cm^−2^, representing significant increments with respect to the reference values. These outcomes far exceed the typical values reported for known 2D materials, suggesting that combined strain/doping engineering may be extended to other 2D materials as a viable method for lattice manipulation. The structural origin of the enhanced polarization with oxygen involvement is further explored by theoretical calculations concerning estimated spontaneous polarization and local charge density.

## 2 Results and Discussion

**Figure 1:**
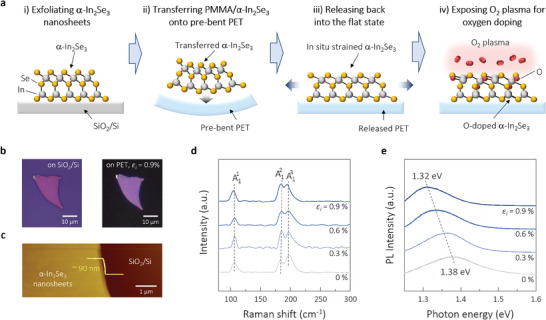
a) Schematic of the critical steps for preparing α‐In_2_Se_3_ nanosheets for the harvesters: i) exfoliating α‐In_2_Se_3_ nanosheets onto SiO_2_/Si, ii) in situ strain engineering during wet transfer of the nanosheets onto a pre‐bent PET, iii) release of the curved nanosheets into a flat state, and iv) treatment of the nanosheet surface with O_2_ plasma. b) Optical micrographs of exfoliated α‐In_2_Se_3_ nanosheets before and after the in situ strain engineering. c) AFM image with a height profile across the interface between the nanosheets and substrate. d) Raman and e) PL spectra of the α‐In_2_Se_3_ nanosheets processed with different in situ strains *ε_i_*.

**Figure** **1**a schematically illustrates the critical steps for preparing piezoelectric energy harvesters based on oxygen‐doped In_2_Se_3_ nanosheets with extra lateral strain induced by a strain‐engineering technique. Each step in the experimental procedure is illustrated in detail in Figure S1 (Supporting Information). Before applying strain engineering, α‐In_2_Se_3_ nanosheets were exfoliated from a bulk specimen and transferred onto a SiO_2_/Si substrate. PMMA was spin‐coated on top of the nanosheets and annealed at 85 °C for 10 min. PMMA/α‐In_2_Se_3_ separated from the substrate, floated onto the water surface, and was finally scooped out onto the center of the PET substrate. In the case of applying the in situ strain *ε_i_*, the PET substrate was bent in a concave manner using the customized fixtures as seen in Figure S2 (Supporting Information). Three different fixtures with controlled curvatures were used to produce different levels of *ε_i_*. Concave bending of the substrate was selected to induce tensile strain in the in‐plane direction in the final α‐In_2_Se_3_ nanosheets after the substrate was released back to the flat state. The extra in‐plane lattice strain is assumed to be beneficial for generating a larger lateral lattice distortion during the bending operation for electromechanical energy conversion.^[^ ^33^, ^41^, ^42^ ^]^ A larger *ε_i_* is induced by a smaller bending curvature, with a maximum tensile *ε_i_* of 0.9% for the smallest curvature of 9.87 mm. The procedure for calculating *ε_i_* with the definition of the neutral plane is presented in Note S1 with Figure S3 (Supporting Information). After chemically etching away the PMMA layer, parallel Ti/Au electrodes (5/50 nm in thickness) were applied to the two ends of the nanosheets by the photolithography process to fabricate energy harvesters. The exposed nanosheet surfaces were then treated in O_2_ plasma atmosphere with controlled exposure times of up to 20 min.

Figure 1b shows optical images of α‐In_2_Se_3_ nanosheet flakes before and after applying the strain engineering with the maximum tensile *ε_i_* = 0.9%. The original shape of the exfoliated sample appears well preserved, with no physical damage in the strain‐engineered sample. Exfoliated nanosheets with a uniform thickness of only 90(±7) nm (corresponding to ≈100 atomic layers) were selected for the energy harvesting evaluation. As shown in the atomic force microscopy (AFM) image in Figure 1c, the thickness was ensured with a line‐profile step of ≈90 nm across the nanosheet/substrate interface. Figure 1d shows Raman spectra with three distinct peaks at ≈106.4, ≈184.1, and ≈197.9 cm^−1^, which are attributed to Raman‐active modes of $A_{1}^{1}$, $A_{1}^{2}$ and $A_{1}^{3}$, respectively,^[^ ^43^, ^44^ ^]^ for α‐In_2_Se_3_ nanosheets processed with *ε_i_* = 0%, 0.3%, 0.6%, or 0.9%. The existence of separated $A_{1}^{2}$ and $A_{1}^{3}$ modes indicates the splitting of longitudinal optical (LO)–transverse optical (TO) modes owing to the lack of inversion symmetry in the *R3m* structure.^[^ ^45^ ^]^ All the vibration peaks shifted slightly toward lower wavenumbers with increasing *ε_i_*, which is associated with elongated chemical bonds because of the larger lateral tensile strain present in the nanosheet.^[^ ^46^, ^47^ ^]^

Figure 1e shows the strain‐dependent photoluminescence (PL) spectra of the α‐In_2_Se_3_ nanosheets. The peak shifts depending on the applied *ε_i_*, i.e., from ≈1.38 eV for unstrained nanosheets to ≈1.32 eV with a maximum *ε_i_* of 0.9%. This red shift indicates a reduction in the optical bandgap owing to the extended bond lengths with the tensile in situ strain.^[^ ^48^, ^49^ ^]^ The bandgap of In_2_Se_3_ is mainly determined by the *sp* hybridization between the In‐*s* and Se‐*p* orbitals and *pp* hybridization between the Se‐*p* orbitals.^[^ ^50^, ^51^ ^]^ A theoretical study suggested that additional tensile strain may lower the conduction band minimum to a greater extent than it decreases the valence band maximum, thereby narrowing the bandgap with tensile strain.^[^ ^51^ ^]^ The apparent shift in the PL emission confirms the incorporation of extra tensile strain into the lattice structure.

**Figure 2:**
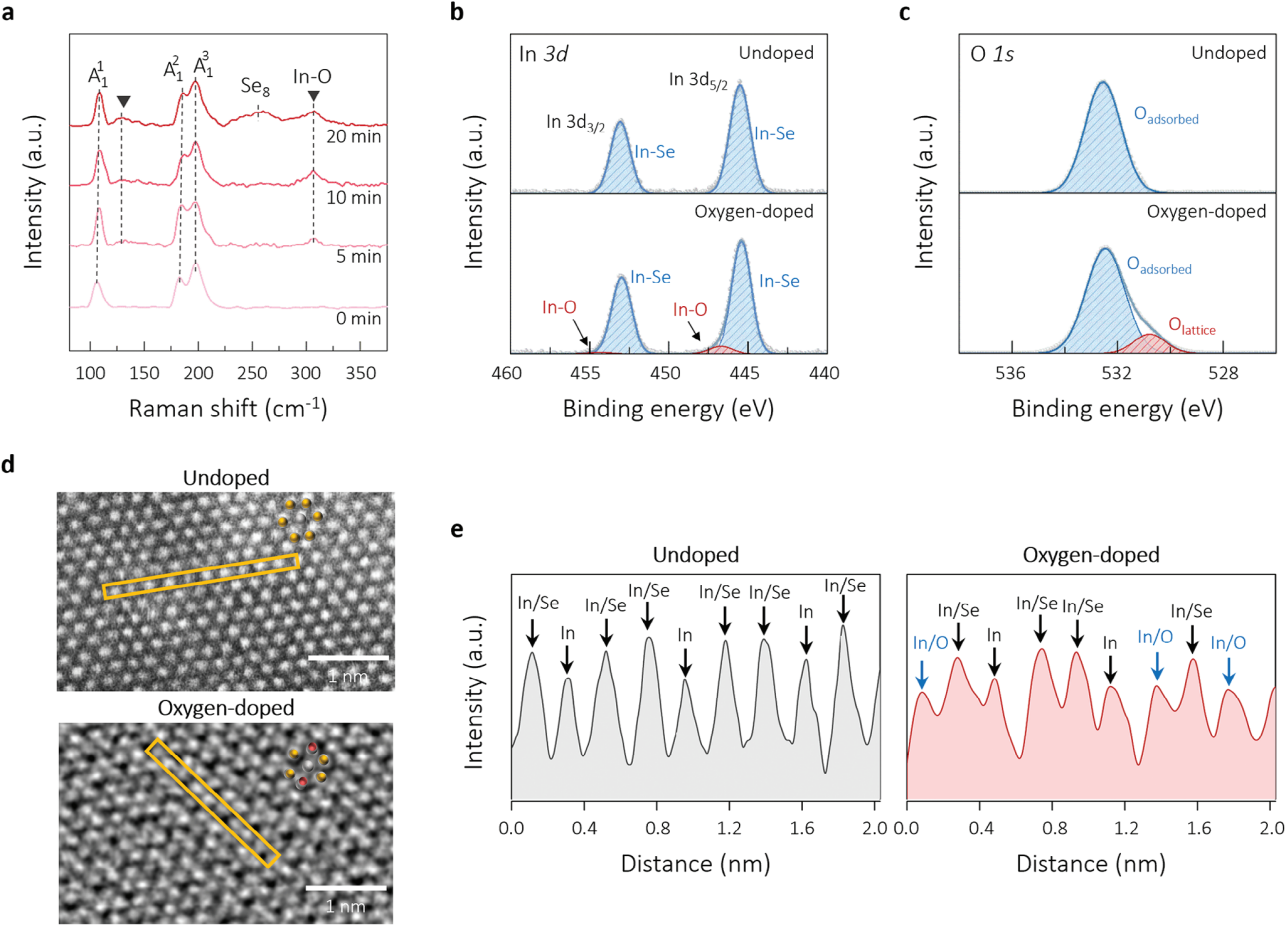
a) Raman spectra of the 0.9%‐strained α‐In_2_Se_3_ nanosheets after plasma treatment for different exposure times of up to 20 min. b) XPS In 3d spectra in the 0.9%‐strained nanosheets before and after the 10 min of plasma exposure, showing the chemical bonds of In─O at ≈446.7 and ≈454.3 eV. c) XPS O 1s spectra in the 0.9%‐strained nanosheets before and after 10 min of plasma exposure, showing evidence of oxygen doping via plasma treatment. d) Atomic resolution STEM‐HAADF images of the undoped and 10min‐doped samples. e) Intensity line profiles of the yellow‐highlighted atomic arrays in the (d) images, demonstrating the substituted oxygen atoms.

The nanosheets strained to a maximum *ε_i_* of 0.9%, were exposed to O_2_ plasma for exposure times of up to 20 min to induce different levels of oxygen doping. **Figure** **2**a shows the variations in the Raman spectra of strained α‐In_2_Se_3_ with different plasma exposure times. In contrast with the spectrum without the O_2_ treatment, additional peaks at ≈130 and ≈304 cm^−1^, which are attributed to the vibration modes of In─O bonds within InSe_6−x_O_x_ octahedral units,^[^ ^52^, ^53^ ^]^ were observed as evidence of oxygen substitutions. The intensity of these peaks increases with the plasma treatment time, indicating the progress of oxygen doping. Another distinct signal at ≈252 cm^−1^, which became more evident with the longer O_2_ treatment, corresponds to the vibration mode of amorphous Se_8_ rings.^[^ ^54^ ^]^ The strong presence of the Se_8_ peak after 20 min of plasma exposure suggests that excessive O_2_ treatment must be avoided to preserve the In_2_Se_3_ structure. This Se_8_ peak was observed when the In_2_Se_3_ sample was processed under harsh conditions such as thermal annealing^[^ ^55^ ^]^ and laser irradiation.^[^ ^56^, ^57^ ^]^ The uniformity of oxygen substitution by the plasma treatment over the nanosheets was ensured by Raman mapping at a specific wavelength of 130 cm^−1^, as shown in Figure S4 (Supporting Information). A uniform color distribution observed after the O_2_ treatment indicates the effective doping of oxygen over the exfoliated sample. The surface of the exfoliated nanosheet was monitored by AFM to confirm the etching level with the extended plasma treatment as seen in Figure S5 (Supporting Information). No significant change in thickness was observed with the progress of plasma exposure although the surface roughness as rms (root mean square) value tended to increase from 1.79 to 3.28 nm after 20 min's exposure.

The X‐ray photoelectron spectroscopy (XPS) analysis was also performed on the plasma‐treated In_2_Se_3_ to confirm the presence of oxygen, as shown in Figure 2b,c. Two distinct main peaks appeared for the In 3d_3/2_ and In 3d_5/2_ states at ≈453.0 and ≈445.4 eV, respectively (Figure 2b), verifying the expected In─Se bonding in In_2_Se_3_.^[^ ^58^ ^]^ For the plasma‐treated sample, additional minor peaks at ≈454.4 and ≈446.7 eV were associated with weak In─O chemical bonding.^[^ ^59^ ^]^ The XPS spectra of the O 1s core levels also confirmed the existence of oxygen in the lattice by demonstrating an additional peak for oxygen in the lattice at ≈530.8 eV, with the major peak at ≈532.6 eV indicating adsorbed oxygen, as shown in Figure 2c.^[^ ^60^, ^61^ ^]^ The atomic ratio between In and Se decreased from 2:3.1 to 2:2.6 with the 10 min oxygen treatment, as roughly estimated by comparing the peak intensities revealed by energy‐dispersive X‐ray spectrometer (EDS) (Figure S6, Supporting Information). We further confirmed the incorporation of oxygen into the Se lattice by performing atomic‐scale scanning transmission electron microscopy (STEM) analysis using the high‐angle annual dark‐field (HAADF) technique as seen in Figure 2d,e. The intensity line profiles of the atomic arrays designated in the images of Figure 2d for the undoped and 10‐min‐doped samples demonstrate the substituted oxygens in the Se sites as a result of the plasma doping.

**Figure 3:**
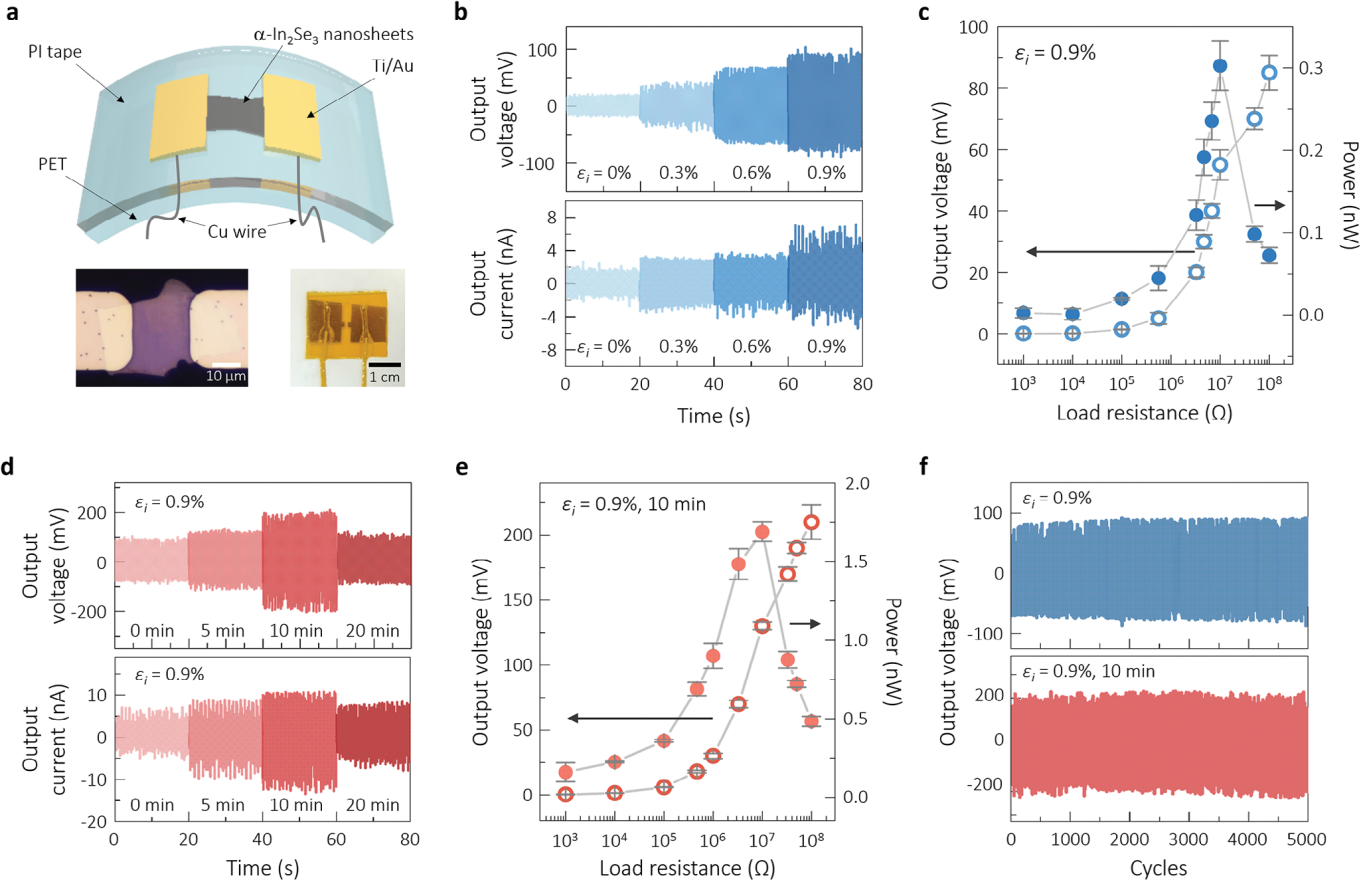
a) Schematic illustration of the structure of the piezoelectric energy harvester consisting of the strained and plasma‐treated α‐In_2_Se_3_ nanosheets and lateral electrodes, along with a top‐view optical micrograph of the nanosheets between the parallel electrodes (left) and an actual photograph of the harvester (right). b) Piezoelectric energy harvesting performance in terms of output voltage and current for the strained‐nanosheet harvesters processed with different in situ strain *ε_i_* of up to 0.9%, measured at a bending frequency of 3 Hz and a bending strain of 2.12%. c) Plots of voltage (left axis) and power (right axis) as functions of the load resistance for the 0.9%‐strained‐nanosheet harvesters. d) Piezoelectric energy harvesting performance of the 0.9%‐strained‐nanosheet harvesters with various plasma exposure times, measured at a bending frequency of 3 Hz and a bending strain of 2.12%. e) Plot of voltage (left axis) and power (right axis) as a function of the load resistance for the 0.9%‐strained and 10‐min‐exposed nanosheet harvesters. f) Output voltage stability of the strained harvesters before and after 10‐min exposure up to 5000 bending cycles.

The effects of the in situ strain and oxygen doping on the piezoelectric power generation of the resulting harvesters were explored using bending operation as the mechanical input source. **Figure** **3**a demonstrates the harvester structure of the α‐In_2_Se_3_ nanosheets on the PET substrate with the lateral Ti/Au electrodes. The nanosheet with a length of ≈20 µm and width of ≈20 µm was carefully positioned in the central region between the neighboring electrodes, as shown in the bottom of Figure 3a. A polyimide (PI) tape was used for device passivation. The bending operation was modulated with variations in bending strain from 1.35% to 2.12% and bending frequencies from 1 to 3 Hz. Figure 3b shows the *ε_i_*‐dependent variations in the output voltage and current of the nanosheet harvesters before the plasma treatment upon repetitive bending at a frequency of 3 Hz and a strain of 2.12%. The measured output values increased significantly with increasing *ε_i_*: the output voltage rose from ≈21.1 mV for *ε_i_* = 0% to ≈103.8 mV for *ε_i_* = 0.9%, while the output current increased from 2.73 to 7.04 nA at the same strain values; thus, applying the maximum in situ strain increased the voltage and current by ≈5 and ≈2.5 times, respectively. The increased outputs for the strained samples are attributed to the extended dipoles along the in‐plane direction upon bending.^[^ ^33^, ^36^ ^]^ The large tensile strain is assumed to create a larger dipole moment and, thus, extra polarization for higher piezoelectric energy conversion. Note that the optimal bending conditions were at 3 Hz and 2.12%, as shown in Figure S7 (Supporting Information), demonstrating the effects of different bending frequencies and strains on the output voltage and current. Figure S8 (Supporting Information) shows the harvesting results with changing polarity, confirming that the harvesting outcomes were due to piezoelectric effects. Figure 3c shows additional plots of voltage and power with the load resistance varying from 1 kΩ to 100 MΩ for the 0.9%‐strained sample. As expected, the voltage increased with the load resistance, whereas the power peaked at a certain resistance. The maximum power was ≈0.30 nW at 10 MΩ, and the peak voltage was ≈90 mV at 100 MΩ.

Figure 3d presents the piezoelectric energy harvesting performance of the 0.9%‐strained sample after the plasma treatment in terms of the output voltage and current with an increasing exposure time of up to 20 min. The plasma treatment improved the harvesting performance depending on the exposure time and, presumably, the doping levels. The highest outputs of ≈210.4 mV and ≈13.5 nA were obtained for the 10‐min‐exposed sample, corresponding to increases of ≈102% and ≈96%, respectively, concerning those of the non‐plasma‐treated sample. The dependence of the load resistance on the voltage and power for the 10‐min harvester is plotted in Figure 3e, which shows that a higher power of ≈1.69 nW was achieved at 10 MΩ after the oxygen treatment. Importantly, optimal oxygen doping increased the power by nearly 5.6 times. The harvesting degradation with excessive treatment for 20 min is likely related to the segregation of Se, as observed in the Raman spectra of Figure 2a. Accordingly, an optimal doping level seems to exist with the limitations of 2D structures in terms of the allowable substitution levels. No comparative studies have been conducted on anion doping and lattice changes for enhancing piezoelectricity. Figure 3f demonstrates the performance stability of the optimized harvester with extended bending up to 5000 cycles for the 0.9%‐strained harvesters before and after 10 min plasma treatment. Fairly consistent voltage values were maintained during the cycling operation in both cases.

**Figure 4:**
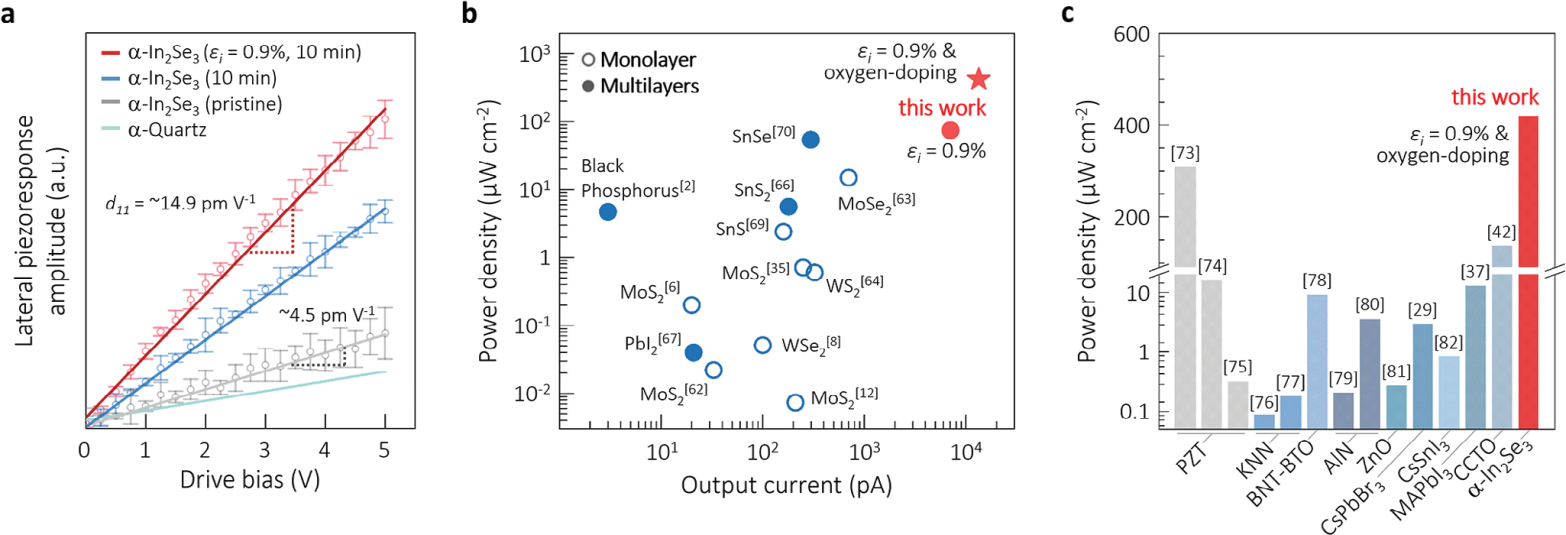
a) Plot of lateral piezoresponse as a function of the drive bias for the undoped and oxygen‐doped α‐In_2_Se_3_ nanosheets with the reference case of α‐quartz, where the steepest slope is attained for the oxygen‐doped strained sample. The slope is converted into the piezoelectric coefficient *d_11_*. b) Projection of our best output current and power density values with the outcomes reported for other 2D materials, demonstrating the excellency of our harvesting results. c) Comparative plots of our best power density with reported values for the representative piezoelectric materials (PZT: Pb(Zr,Ti)O_3_, KNN: (K,Na)NbO_3_, BNT‐BTO: ((Bi,Na)Ti)O_3_‐BaTiO_3_, MA: methylammonium, and CCTO: CaCu_3_Ti_4_O_12_).

Piezoelectricity of the α‐In_2_Se_3_ samples was evaluated using the plots of the lateral piezoresponse with respect to the applied drive bias as a result of the AFM measurements in the lateral mode as shown in **Figure** **4**a. The resultant lateral piezoelectric coefficient *d_11_* values were obtained from the slopes of the plots for the α‐In_2_Se_3_ samples treated in various conditions. Note that our harvester works under the lateral tensile strain with the bending operation. Extended information on *d_11_* for different plasma conditions is available in Figure S9 (Supporting Information). As expected, the *d_11_* value was substantially increased from ≈4.5 to ≈10.3 pm V^−1^ until the exposure time of 10 min, indicating the positive effect of oxygen substitutions on the extension of the dipole moment in the lateral direction in the In_2_Se_3_ nanosheets. The best *d_11_* of 14.9 pm V^−1^ was achieved for the optimized sample subjected to the 0.9% in situ strain with the 10 min exposure, representing a 3.3‐fold increase compared to the untreated sample. As seen in Figure 4b, our best current and power density values of ≈13.5 nA and ≈420 µW cm^−2^ were projected onto a plot for comparison with the harvesting values reported for other 2D materials.^[^ ^2^, ^6^, ^8^, ^10^, ^12^, ^28^, ^35^, ^62^, ^63^, ^64^, ^65^, ^66^, ^67^, ^68^, ^69^, ^70^, ^71^, ^72^ ^]^ The detailed information on 2D materials, dimensions, and harvesting outcomes is available in Table S1 (Supporting Information). Our values are far better than other reported outcomes, indicating that the strain/doping engineered α‐In_2_Se_3_ is very competitive in generating power with superior piezoelectricity. Even the best power density of ≈420 µW cm^−2^ is better than the density values reported for other representative piezoelectric materials such as Pb(Zr,Ti)O_3_ and (K,Na)NbO_3_ as compared in Figure 4c (with the values listed in Table S2, Supporting Information).^[^ ^29^, ^37^, ^42^, ^73^, ^74^, ^75^, ^76^, ^77^, ^78^, ^79^, ^80^, ^81^, ^82^ ^]^ As far as we know, the reported best power density value is ≈308 µW cm^−2^ for a 2.8‐µm‐thick PZT film sputter‐deposited on a stainless steel substrate.^[^ ^73^ ^]^

**Figure 5:**
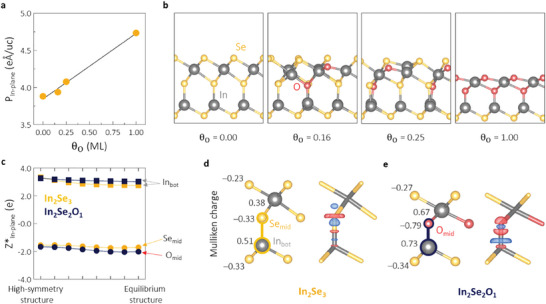
a) Calculated in‐plane polarization P_in‐plane_ of the oxygen‐doped In_2_Se_3_ systems as a function of oxygen surface coverage θ_O_. b) Side views of the atomic structures of a monolayer of In_2_Se_3_ with different oxygen surface coverages from 0.00 to 1.00 ML. In, Se, and O atoms are represented by gray, yellow, and red spheres, respectively. (c) In‐plane dynamic charges Z^*^_in‐plane_ for In_bot_, Se_mid_, and O_mid_ from the high‐symmetry structure to the equilibrium structure in In_2_Se_3_ (yellow) and In_2_Se_2_O_1_ (navy). In_bot_: In in the bottom layer of In (in In_2_Se_3_ and In_2_Se_2_O_1_); Se_mid_: Se in the middle layer of Se (in In_2_Se_3_); and O_mid_: O in the middle layer of O (in In_2_Se_2_O_1_). The calculated Mulliken charges and the corresponding atomic structure models are shown in (d,e), with the In─Se bond highlighted in yellow for (d) In_2_Se_3_ and the In─O bond highlighted in navy for (e) In_2_Se_2_O_1_. The corresponding difference in charge density Δρ(r) is shown in (d,e), where the red and blue regions denote the accumulation and depletion of electron density, respectively, with an isosurface value of 0.007e Å^−3^.

As an extra effort, the origin of the enhancement due to oxygen doping was explored using a theoretical simulation of the structural changes and polarization. **Figure** **5**a shows the results of analyzing how the O_2_ plasma affects the polarity of In_2_Se_3_ through the density functional theory (DFT) calculations, revealing that the in‐plane polarization P_in‐plane_ increases with the surface oxygen coverage θ_O_. The surface oxygen coverage simulates the gradual progress of oxygen substitution in the In_2_Se_3_ structures, where the coverage is defined as the ratio of the number of oxygen substitution sites to the number of anion sites in the topmost and middle layers. In this context, the exposure time in the experiment corresponds to the oxygen concentration in the theoretical model. The more detailed procedure for the modeling of the incorporation of oxygen as an impurity into α‐In_2_Se_3_ is described in the Experimental Section. The increase in P_in‐plane_ with θ_O_ was consistent with the trend in the experimentally measured lateral piezoelectric response. Thus, the increase in the polarized charge densities is expected to significantly influence the piezoelectric effects, as previously reported for the α‐In_2_Se_3_ system, where the increasing polarization via the rearrangement of the dipole orientations strengthens the piezoelectric effect.^[^ ^28^, ^83^ ^]^ Figure 5b shows the lowest energy structures for each surface oxygen coverage and the defect formation energies of all symmetrically inequivalent configurations for each oxygen coverage are tabulated in Table S3 (Supporting Information). See Note S2 (Supporting Information) for the procedure of energy calculation and Figure S10 (Supporting Information) for more detailed schematics of substitution configurations. To gain more insight into the mechanism underlying the enhanced in‐plane polarization, the dynamic charges Z^*^, defined as the rate of change in polarization with atomic displacement, were further investigated in relation to the behavior of the local dipole moment.^[^ ^84^, ^85^ ^]^ It has been experimentally and theoretically verified that the central Se atom in α‐In_2_Se_3_ contributes critically to the electric dipole.^[^ ^27^, ^86^ ^]^ Therefore, to facilitate a comprehensive analysis of the effect of O substitution on the local dipole behavior, we compared the local dipole moment behavior of pristine In_2_Se_3_ and the In_2_Se_2_O_1_ system, in which the Se atom was substituted by an O atom only in the middle layer.

Figure 5c shows the dynamic charges in the in‐plane direction (Z^*^_in‐plane_) for the bottom layer of In (designated In_bot_) and the middle layers of Se (Se_mid_) and O (O_mid_) in In_2_Se_3_ and In_2_Se_2_O_1_. Of prime importance is that the dynamic charges of O at equilibrium for In_2_Se_2_O_1_ are larger (−2.02e) than those of Se in In_2_Se_3_ (−1.70e), thus enhancing the in‐plane polarization in oxygen‐doped In_2_Se_3_. The larger dynamic charge values of O compared with Se can be understood by the larger electronegativity of O, which drives the In cation to donate its electrons to the O anion more readily than it does to the Se anion. This is rationalized by the calculated Mulliken charges and the corresponding differences in charge density (Figure 5d,e), illustrating the electronic charge transfer from the In cations to the Se (or O) anions.^[^ ^87^, ^88^ ^]^ Our results demonstrate that the O anion in In_2_Se_2_O_1_ bears a more negative charge (−0.79e; Figure 5e, labeled navy) than the Se anion in In_2_Se_3_ (−0.33e; Figure 5d, yellow). Meanwhile, the contributing In cation in In_2_Se_2_O_1_ has a more positive charge (+0.73e; Figure 5e, navy) than that in In_2_Se_3_ (+0.51e; Figure 5d, yellow). This is nicely confirmed by the volumetric charge density differences Δρ(r) visualized in Figure 5d,e, where more electrons are accumulated around the O anion than around the Se anion, whereas the opposite tendency is observed in the case of the In cations. These DFT results strongly support our experimental observations and demonstrate that the polarized charge densities increased during the O_2_ plasma treatment, which had an influence on the observed improvement in the electromechanical performance.

**Figure 6:**
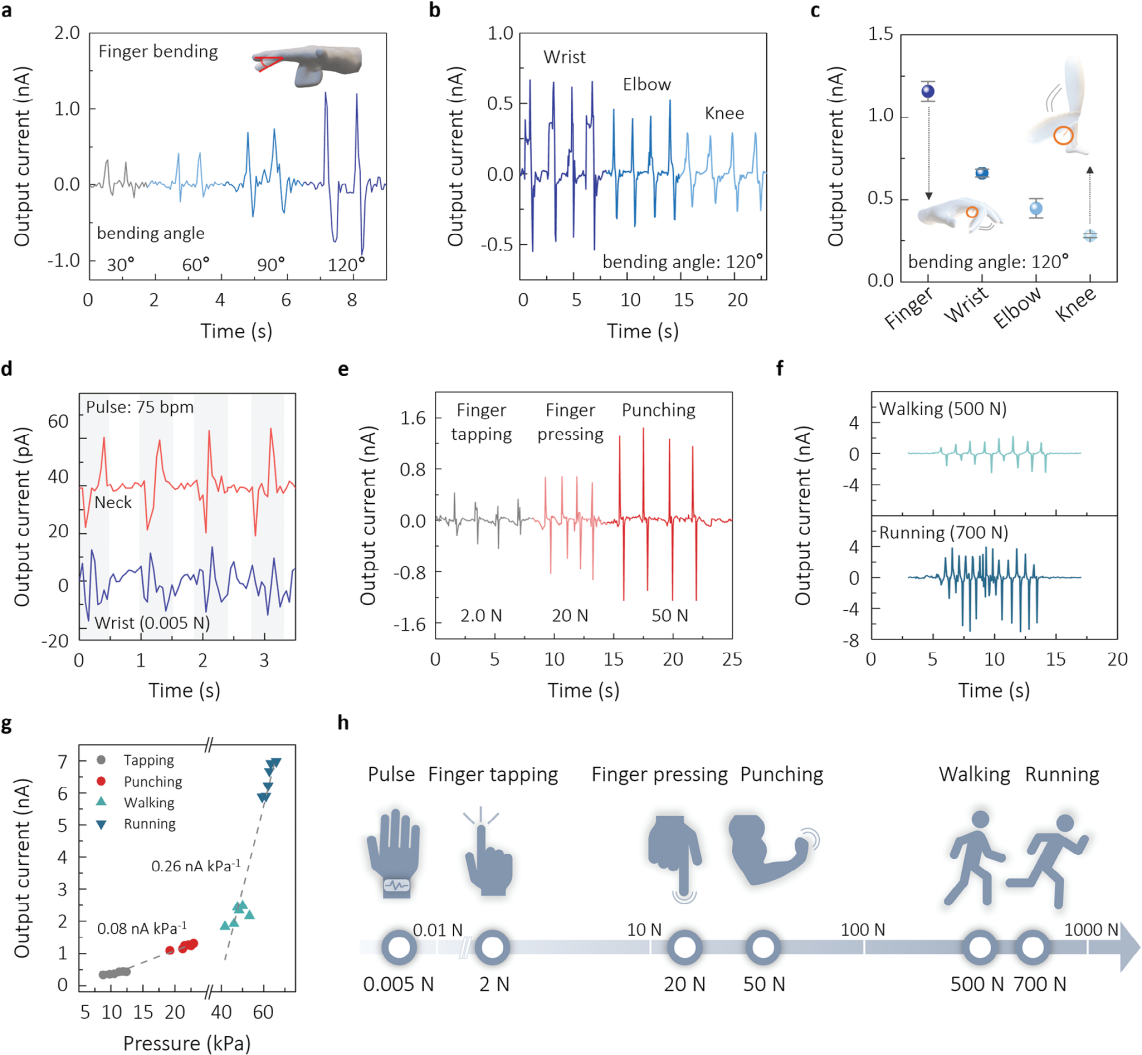
a) Output current signals of the optimal α‐In_2_Se_3_ harvesters in response to finger‐bending with different bending angles. b) Output current generated from various joints in human body including wrist, elbow, and knee for the attached devices. c) Plot of electrical signals per each bending motion, measured at the identical bending angle of 120°. d) Performance of the α‐In_2_Se_3_ harvesters as real‐time pulse sensors attached to the neck and wrist. e,f) Output currents generated by finger‐tapping, finger‐pressing, and punching (e), and walking and running (f). g) Plot of output current versus applied pressure generated from the motions in (e,f), with the estimated sensitivity values from the slopes in the low‐ and high‐pressure regions. h) Schematic illustration of a covered force range for detecting various physiological motions using the α‐In_2_Se_3_ device.

The physiological sensing abilities of the optimally conditioned α‐In_2_Se_3_ harvester were further evaluated by collecting electrical signals from different mechanical input sources created by diverse human motions. **Figure** **6**a,b demonstrates the output currents generated by various motions of the human body, which represent the capability of detecting the bending motions. For the bending tests, the electromechanical device was attached to the finger, wrist, elbow, or knee to monitor real‐time joint movements in electrical signals. Figure 6a shows the effect of the angles of finger bending on the piezoelectric outputs: the generated currents increased from ≈320 pA to ≈1.22 nA with varying bending angles from ≈30° to ≈120c. The repetitive bending operations of the identical device placed on the wrist, elbow, or knee also produced distinct output signals depending on the type of bending motion as shown in Figure 6b. Figure 6c presents the output values produced at the same bending angle of 120° for different human body motions, which indicates that the smaller bending radius tends to produce the larger output signal. For instance, the finger bending with the smaller curvature than that for the knee bending exhibited the larger current value.

The α‐In_2_Se_3_ harvester is also applicable as a piezoelectric pressure sensor for monitoring instantaneous compressive forces. Figure 6d–f demonstrates examples of detecting a wide range of forces generated by human pulses (corresponding to a force of ≈0.005 N) to running motions (≈700 N).^[^ ^89^ ^]^ With the device attached onto the skin surfaces of the wrist and neck, regular pulse signals of ≈10 to ≈20 pA were detected with the higher currents on neck (Figure 6d). The consistent pulse rate of 75 bpm (beat per minute) was ensured from regular signals produced by both the wrist and neck pulses. Figure 6e shows the different levels of output currents generated by the motions of finger‐tapping (≈2 N), finger‐pressing (≈20 N), and punching (≈50 N). The magnitudes of the input forces were measured using a commercial force sensor. As the electrical output is directly proportional to the magnitude of the mechanical input source, the lowest current of 0.43 nA was obtained for the finger‐tapping and the highest of 1.31 nA for punching. The walking (≈500 N) and running (≈700 N) motions also created clear output currents of ≈2.48 and ≈6.92 nA, respectively, as seen in Figure 6f. The electrical output values per each mechanical input pressure were plotted as seen in Figure 6g to attain the sensitivity from the slopes of the correlated lines.^[^ ^41^, ^90^ ^]^ The sensitivities were 0.08 and 0.26 nA kPa^−1^ in the low‐ and high‐pressure regions, respectively, which are very competitive to previously reported values for piezoelectric sensors based on representative piezoelectric materials including 2D transition metal dichalcogenides,^[^ ^91^ ^]^ Nylon‐11,^[^ ^92^ ^]^ halide perovskite,^[^ ^93^ ^]^ and ZnO.^[^ ^94^, ^95^ ^]^ Conclusively, our optimal device can be used successfully as physiological sensors covering the wide range of human motions from ≈0.005 to ≈700 N with excellent sensitivities as schematically illustrated in Figure 6h.

## 3 Conclusion

We successfully demonstrated an effective approach combining strain engineering and oxygen doping to improve the piezoelectric properties of α‐In_2_Se_3_ nanosheets. The in situ strain‐engineering process initially facilitated lattice extension in the lateral direction through deliberately induced tensile strain. Subsequent oxygen plasma doping, involving the substitution of selenium atoms with oxygen on the surface of the nanosheets, led to larger lattice distortion and polarization enhancement. Theoretical calculations of in‐plane polarization and the local dipole moment behavior corroborated the structural origins of this anion doping‐induced enhancement, revealing that the polarized charge densities increased during the oxygen doping due to the larger dynamic charge values and electronegativity of O compared to Se. The optimally conditioned device achieved notable improvements in piezoelectric energy harvesting performance, with output levels reaching ≈13.5 nA and ≈420 µW cm^−2^, significantly surpassing the values reported for existing 2D piezoelectric materials. Our findings demonstrate that the integration of anion doping and strain engineering is a robust strategy for enhancing the piezoelectric properties of 2D materials.

## 4 Experimental Section

### Preparation of Strained α‐In_2_Se_3_ Nanosheets

Extra lattice strain was imposed in α‐In_2_Se_3_ nanosheets by a unique strain engineering method. Specifically, α‐In_2_Se_3_ nanosheets were exfoliated from a bulk α‐In_2_Se_3_ sample (HQ Graphene) using a regular 3M adhesive tape and transferred onto a SiO_2_/Si substrate. The substrate was initially cleaned by ultrasonication sequentially in acetone, isopropanol, and deionized water, followed by O_2_ plasma treatment at 100 W for 10 min. Next, a PMMA (950 PMMA A6, Micro Chem.) solution was spin‐coated onto the α‐In_2_Se_3_/SiO_2_/Si at 3000 rpm for 1 min and annealed at 85 °C for 10 min on a hot plate. The PMMA/α‐In_2_Se_3_ specimen was separated from SiO_2_/Si by immersion in a 0.1 m NaOH solution at 85 °C for 10 min. The separated PMMA/α‐In_2_Se_3_ floated onto the surface of a water bath and was then scooped up by a flat or bent PET substrate. The curvature of the concavely bent PET substrate was controlled with three home‐made fixtures having different curvatures to induce the in‐plane tensile strains of 0.3, 0.6, and 0.9% in the α‐In_2_Se_3_ nanosheets when the bent PET was released back to the flat state. The resulting PMMA/α‐In_2_Se_3_/PET specimen was dried at 65 °C for 3 h and released from the holder. The PMMA was dissolved entirely in acetone for 15 min and rinsed several times with deionized water.

### Fabrication of Piezoelectric Energy Harvester with Oxygen Doping

Piezoelectric energy harvesters consisting of α‐In_2_Se_3_ transferred onto a PET substrate were fabricated with two parallel side electrodes. Photolithography was carried out to pattern the electrodes by sequentially spin‐coating the lift‐off (LOR‐2A, Kayaku Advanced Materials) and photoresist (AZ GXR‐601, AZ Electronic Materials) layers prior to light exposure. After the developing process using a developer (AZ 300 MIF, AZ Electronic Materials), thermal evaporation was conducted for the deposition of Ti/Au (5/50 nm thick). The photoresist pattern was finally dissolved in acetone for 15 min. The O_2_ plasma treatment was conducted at 100 kHz with a power of 100 W under a gas flow rate of 40 sccm on the exposed area of the electrode‐patterned harvester. The doping levels were controlled by changing exposure time from 0 to 20 min. For the electrical connection, Cu wires were externally attached to the electrodes. Finally, the plasma‐treated device was sealed with a PI tape for passivation.

### Characterization and Measurement

The crystallinity and PL properties of the exfoliated sample were examined using a Raman spectrometer (Invia RE04, Renishaw) with a 532‐nm laser excitation source. The reference Si peak at 520 cm^−1^ was used for calibration of Raman spectra. The surface topology and thickness of the samples were characterized by AFM (NX‐10, Park Systems) in non‐contact mode. XPS (K‐alpha, Thermo VG) was conducted using an Al Kα radiation source (1486.6 eV) in an ultrahigh‐vacuum chamber at 1.0 × 10^−8^ Torr to examine the chemical states of the samples. The atomic ratio between In and Se was roughly estimated using a field‐emission scanning electron microscope (FE‐SEM; JSM‐7610F‐Plus, JEOL) equipped with an EDS. HAADF‐STEM images of the plasma‐treated samples were obtained using a Cs‐corrected STEM unit (JEM‐ARM200F, JEOL) under an accelerating voltage of 80 kV. The samples for the STEM observation were prepared by the PMMA‐assisted wet transfer method onto Cu grids. A nanovoltmeter (2128A, Keithley) and galvanostat system (IviumStat, Ivium Technologies) were used to measure the generated voltage and current of the resultant harvesters. The piezoelectric characteristics were examined by piezoresponse force microscopy (PFM; XE‐100, Park Systems) to determine the lateral piezoelectric coefficient *d_11_* using a nonconductive silicon cantilever tip (Multi 75‐G, Budget Sensors) in contact mode. The lateral piezoresponse signal was recorded using a lock‐in amplifier (SR830, Stanford Research Systems).

### Estimation by Density Functional Theory

All calculations were performed using DFT with the projector augmented wave (PAW) method, as implemented in the Vienna Ab initio Simulation Package (VASP).^[^ ^96^, ^97^, ^98^ ^]^ The Kohn–Sham orbitals were expanded using a plane‐wave basis set, and the kinetic cutoff energy was set to 600 eV. The Brillouin zone was sampled via a Γ‐centered k‐point mesh with an equivalent k‐spacing of 0.20 Å^−1^. The generalized gradient approximation of the exchange‐correlation functional (optB86b) was used with van der Waals (vdW) corrections via the self‐consistent nonlocal vdW‐correction scheme.^[^ ^99^, ^100^ ^]^ A vacuum region (in the out‐of‐plane direction) of 20 Å was adopted to mitigate the nonphysical interactions between the periodically repeated images. Dipole correction^[^ ^101^ ^]^ was included in the calculations to adjust the misalignment arising from the intrinsic electric polarization between the vacuum levels on opposing sides of α‐In_2_Se_3_ due to the asymmetric structure. All geometries were fully optimized until the maximum Hellmann–Feynman force was smaller than 10^−3^ eV Å^−1^. The electric polarization was evaluated by using the following approximation: $P_{s}=\;\left(e/\Omega \right)\sum_{i}Z_{i}^{*}\delta d_{i}$, where *i* denotes the *i*th atom, $Z_{i}^{*}$ was the Born effective charges associated with the atomic displacement (δ*d_i_*) of the *i*th atom from their position in the unpolarized structure, *e* was the charge of an electron, and Ω was the cell volume considered.^[^ ^84^, ^102^, ^103^, ^104^, ^105^ ^]^ By comparing the reference non‐polar structure and the ferroelectric one, the atomic displacements responsible for ferroelectricity can be analyzed. For the Born effective charge $Z^{*}$ of each atom, as calculated by density functional perturbation theory (DFPT), a rectangular orthorhombic supercell was applied for the linear response with respect to external fields commensurate with the supercell. The Mulliken charge analysis was performed using the local orbital basis suite and electronic structure reconstruction (LOBSTER) code.^[^ ^106^, ^107^ ^]^

To mimic the experiments and understand how the O_2_ plasma treatment affects the polarity in In_2_Se_3_, the incorporation of oxygen into monolayer α‐In_2_Se_3_ was modeled with the surface oxygen coverage varying from 0.16 to 1.00 monolayer (ML) by constructing 1 × 1 × 1, 2 × 2 × 1, and 3 × 3 × 1 supercells based on the hexagonal primitive unit cell, where the coverage was defined as the ratio of the number of oxygen‐substituted sites to the number of anion sites in the topmost and middle layers. Here, the exposure time in the experiment corresponded to the oxygen concentration in the theoretical model. All symmetric inequivalent configurations with different oxygen surface coverages were constructed using the Supercell code.^[^ ^108^ ^]^ For these structures, substitutional oxygen atoms were introduced into the topmost and middle layers of Se, and the bottom layer of Se was not considered a doping site to mimic the bulk region; it was also fixed during geometry optimization. The initial lattice constants of the oxygen‐incorporated In_2_Se_3_ were fixed using the optimized values of the corresponding pure In_2_Se_3_ supercell, and then, only the atomic positions were relaxed. Symmetrically inequivalent configurations with different (surface) oxygen coverages ranged from 0.16 to 1.00 ML. The configuration with the lowest energy was selected for each oxygen coverage for further polarization calculations.

## Conflict of Interest

The authors declare no conflict of interest.

## Author Contributions

J.Y.K., W.H., S.Y.H., and Y.S.J. contributed equally to this work.

## Supporting information



Supporting Information

## Data Availability

The data that support the findings of this study are available from the corresponding author upon reasonable request.
