# Supplementary material for: Oxygen‐Doped 2D In2Se3 Nanosheets with Extended In‐Plane Lattice Strain for Highly Efficient Piezoelectric Energy Harvesting
Source: Adv Sci (Weinh). 2024 Nov 26;12(3):2410851. doi: 10.1002/advs.202410851 (PMC11744569; doi:10.1002/advs.202410851)
Supplement: Supplementary file 1 — Supporting Information [file ADVS-12-2410851-s001.docx]

Supporting Information

**Oxygen-Doped 2D In_2_Se_3_ Nanosheets with Extended In-Plane Lattice Strain for Highly Efficient Piezoelectric Energy Harvesting**

Ji Yeon Kim^#^, Woohyun Hwang^#^, Seo Yeon Han^#^, Ye Seul Jung^#^, Fengyi Pang, Wenhu Shen, Cheolmin Park, Sang-Woo Kim^*^, Aloysius Soon^*^, and Yong Soo Cho^*^

J. Y. Kim, W. Hwang, S. Y. Han, Y. S. Jung, F. Pang, W. Shen, Prof. C. Park, Prof. S.-W. Kim, Prof. A. Soon, Prof. Y. S. Cho

Department of Materials Science and Engineering, Yonsei University, Seoul 03722, Republic of Korea

E-mail: kimsw1@yonsei.ac.kr, aloysius.soon@yonsei.ac.kr, ycho@yonsei.ac.kr

Y. S. Jung

SK Hynix, Icheon, Gyeonggi-do 17336, Republic of Korea


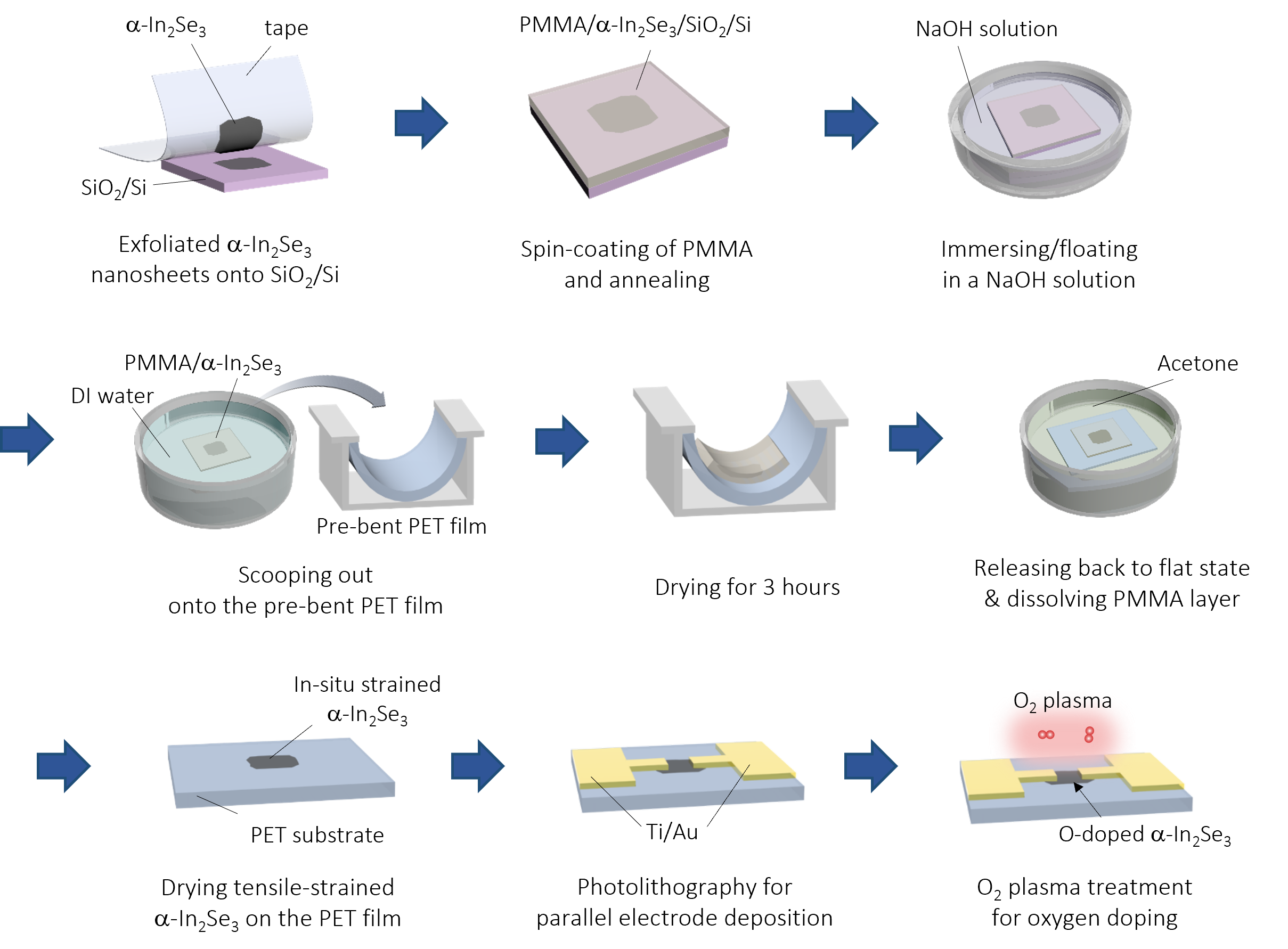


**Figure S1.** Schematic procedure for the preparation of harvester samples starting from exfoliation of the In_2_Se_3_ nanosheets. The step-by-step processes illustrate the sequence of applying the in situ straining and anion-doping techniques to impose extra lattice strain in α-In_2_Se_3_ nanosheets on a PET substrate.


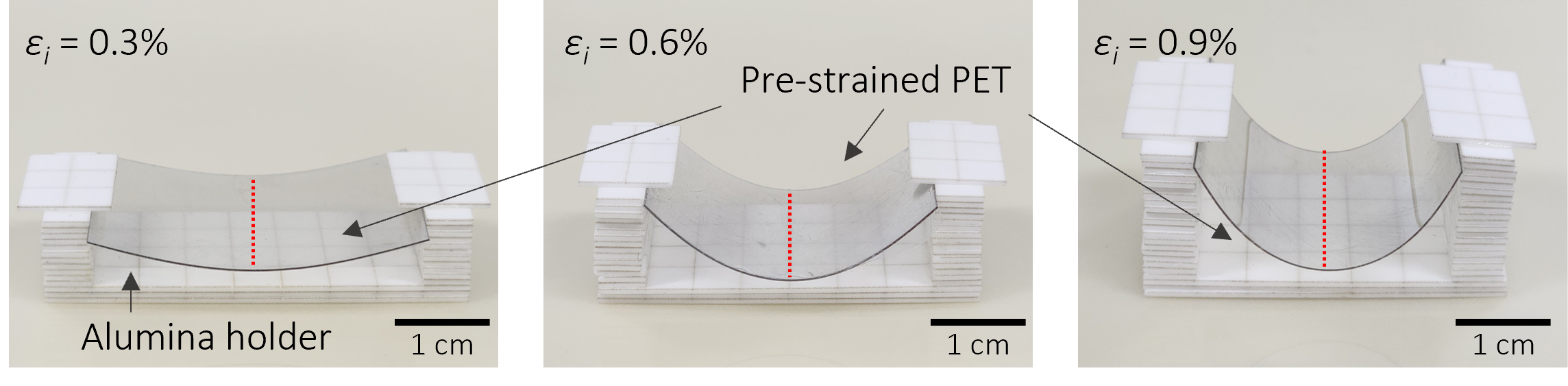


**Figure S2.** Photographs of the fixtures for pre-straining the exfoliated samples, which have different curvatures for different levels of in situ strain *ε_i_*.

**Note S1**: Estimation of the neutral plane and applied in situ strain


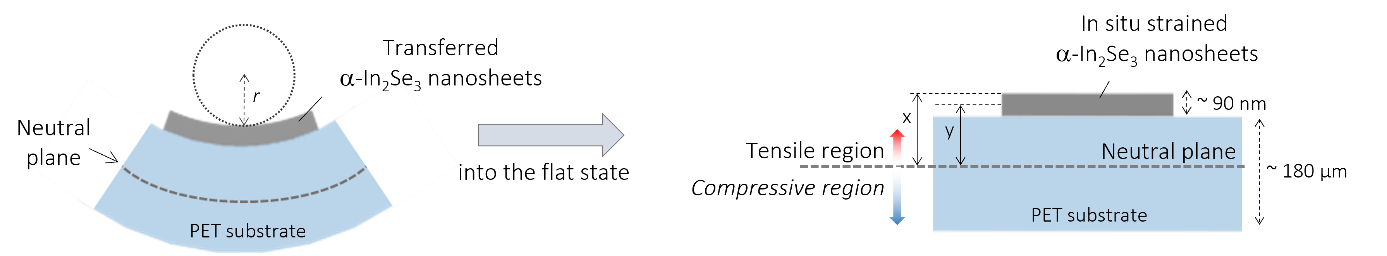


**Figure S3.** Schematic illustration of the location of neutral plane in the α-In_2_Se_3_/PET structure, which was used for the calculation of in situ strain.

*1) Estimation of the neutral plane*

The position of the neutral plane, *x*, related to the top surface was calculated to estimate the strain imposed in situ on α-In_2_Se_3_ considering the contribution of each layer in the α-In_2_Se_3_/PET structure using the following equation (*Science* **2009**, *325*, 977):

$x=\frac{E_{In2Se3}^{*}t_{In2Se3}\left( t_{In2Se3}-\frac{t_{In2Se3}}{2} \right)+E_{PET}^{*}t_{PET}\left( t_{In2Se3}+t_{PET}-\frac{t_{PET}}{2} \right)}{E_{In2Se3}^{*}t_{In2Se3}+E_{PET}^{*}t_{PET}}$ (1)

where *E** = *E*/(1 − *v^2^*) (here, *E* and *v* are Young’s modulus and Poisson’s ratio of each layer, respectively), and *t* is the thickness of each layer.

The following data were used for the calculation:

*E_In2Se3_* = 105.5 GPa, *ν_In2Se3_* = 0.31 for α-In_2_Se_3_ nanosheets (*Inorg. Mater.* **2011**, *47*, 1174)

*E_PET_* = 3.1 GPa, *ν_PET_* = 0.43 for the 0.18-mm-thick PET substrate (*Materials* **2016**, *9*, 850)

The neutral plane *x* was found to be 0.088869 mm.

*2) Calculation of the in situ strain in the harvester*

The in situ strain *ε_i_* applied in α-In_2_Se_3_ nanosheets was calculated using the relation *ε_i_ = y/r*, where *y* is the distance from the neural plane (*x*) to the middle of the α-In_2_Se_3_ nanosheet and *r* is the radius of bending curvature, as depicted in Figure S3. The *r* value was calculated using the following equation (*Mater. Horizons* **2022**, *9*, 1207):

$r= \frac{L}{2\pi\sqrt{\frac{\Delta L}{L}-\frac{\pi^{2}{t_{tot}}^{2}}{12L^{2}}}}$ (2)

where *L* is the substrate length, *ΔL* is the reduced length of the pre-bent substrate, and *t_tot_* is the total thickness of α-In_2_Se_3_ and PET. The calculated values are as follows:

| **Substrate length**  ***L* (mm)** | **Reduced length**  ***ΔL* (mm)** | **Radius of curvature**  ***r* (mm)** | **In situ strain**  ***ε_i_* (%)** |
| --- | --- | --- | --- |
| 36.4 | 1.39 | 29.61 | 0.3 |
| 34.7 | 4.83 | 14.80 | 0.6 |
| 32.6 | 9.0 | 9.87 | 0.9 |


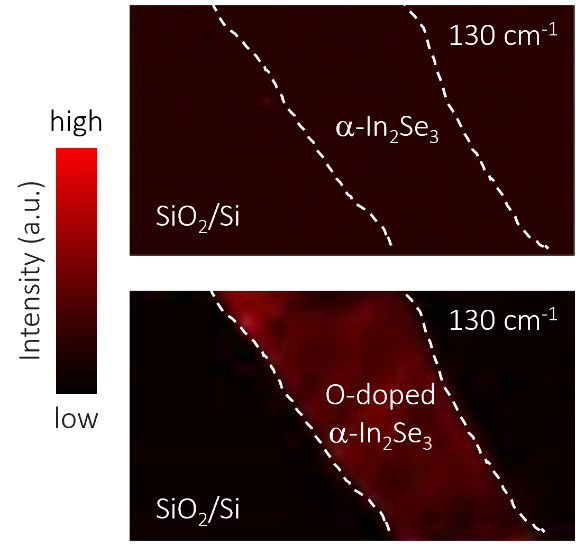


**Figure S4.** Raman mapping images at a specific wavelength of 130 cm**^−^**^1^ for the 0.9%-strained nanosheets before and after 10 min of plasma exposure, indicating a stronger intensity in the α-In_2_Se_3_ sample at the wavelength after the plasma treatment.


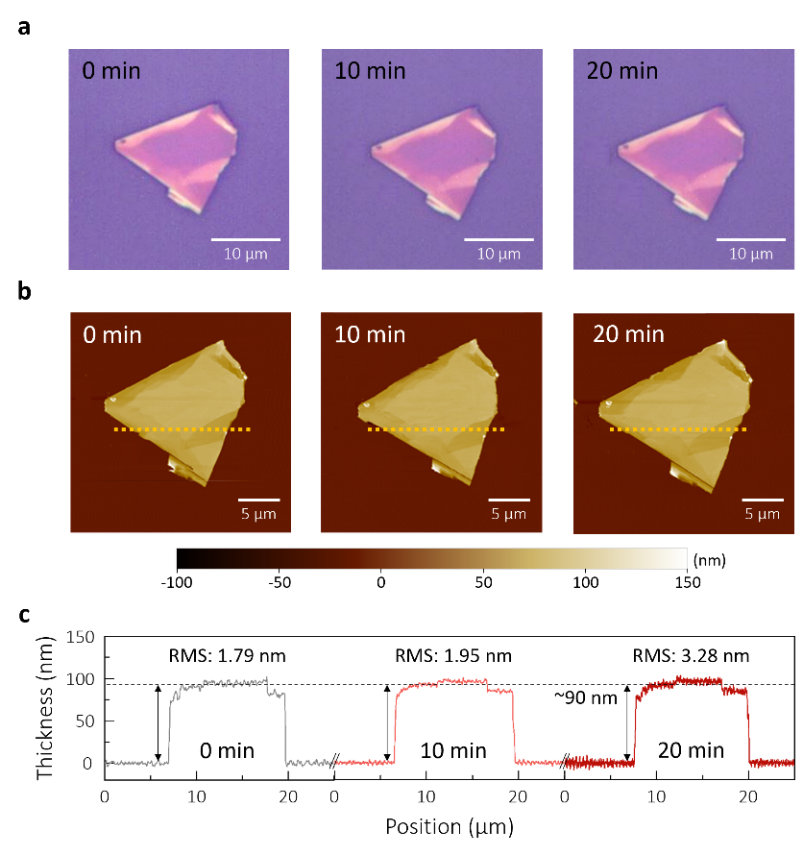


**Figure S5.** (a,b) AFM images of an exfoliated In_2_Se_3_ nanosheet with the progress of O_2_ plasma treatments for 0, 10, and 20 min, demonstrating no etching effect (or thickness change) with the plasma treatment. (c) Changes in rms roughness in the In_2_Se_3_ nanosheet with the plasma exposure times.


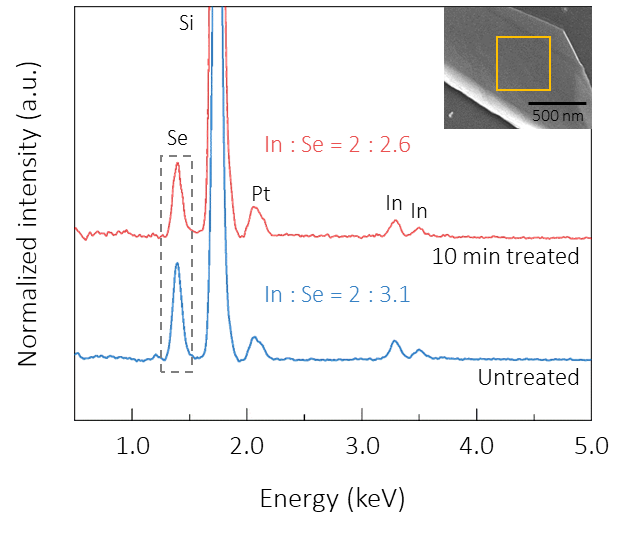


**Figure S6.** EDS spectra of 0.9%-strained nanosheets before and after 10 min’s plasma exposure for the designated area of the inset SEM image, suggesting the change in the ratio of In:Se after the plasma treatment.


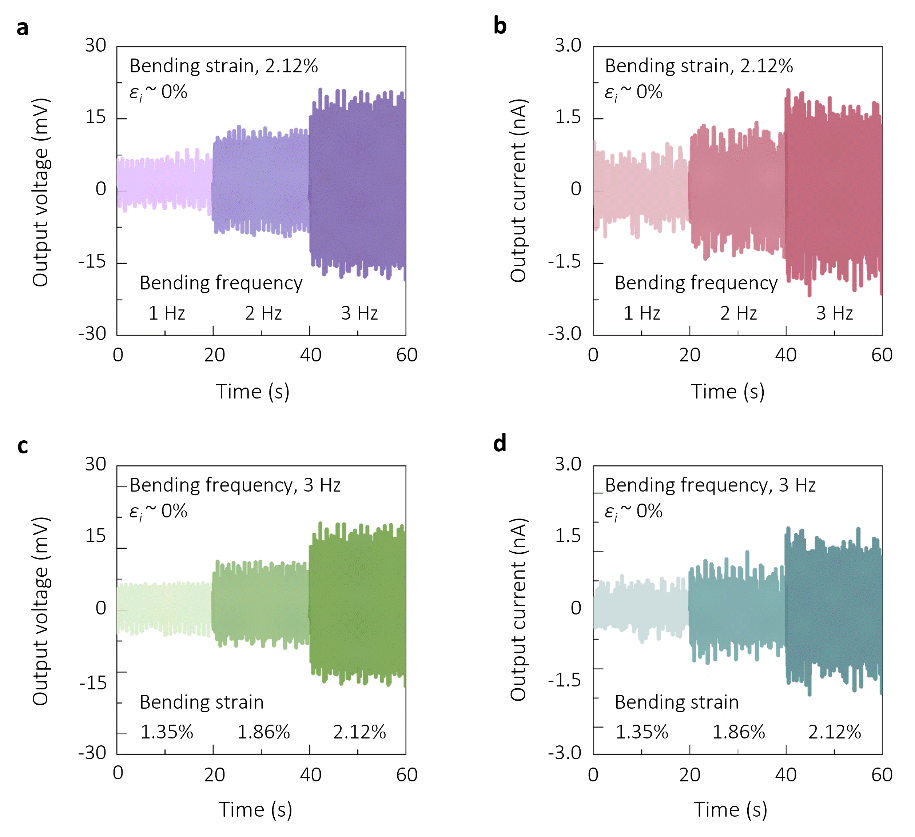


**Figure S7.** Piezoelectric energy harvesting performance of unstrained α-In_2_Se_3_ nanosheets: (a) output voltage and (b) output current with the change in bending frequency from 1 Hz to 3 Hz under a 2.12% bending strain, and (c) output voltage and (d) output current with the change in bending strain from 1.35% to 2.12% under a 3 Hz bending frequency.


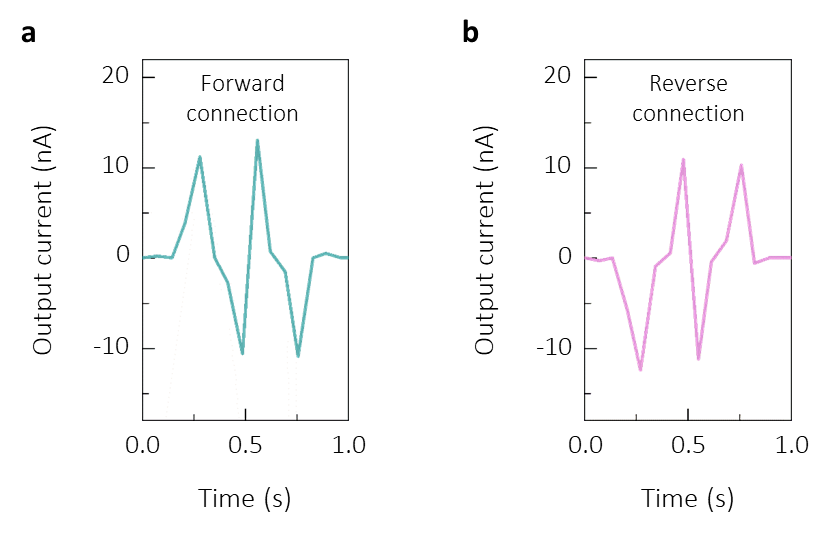


**Figure S8.** Polarity-switching behavior with (a) forward and (b) backward connections, which confirms that the output currents were generated by the piezoelectric effect of the α-In_2_Se_3_ nanosheets.


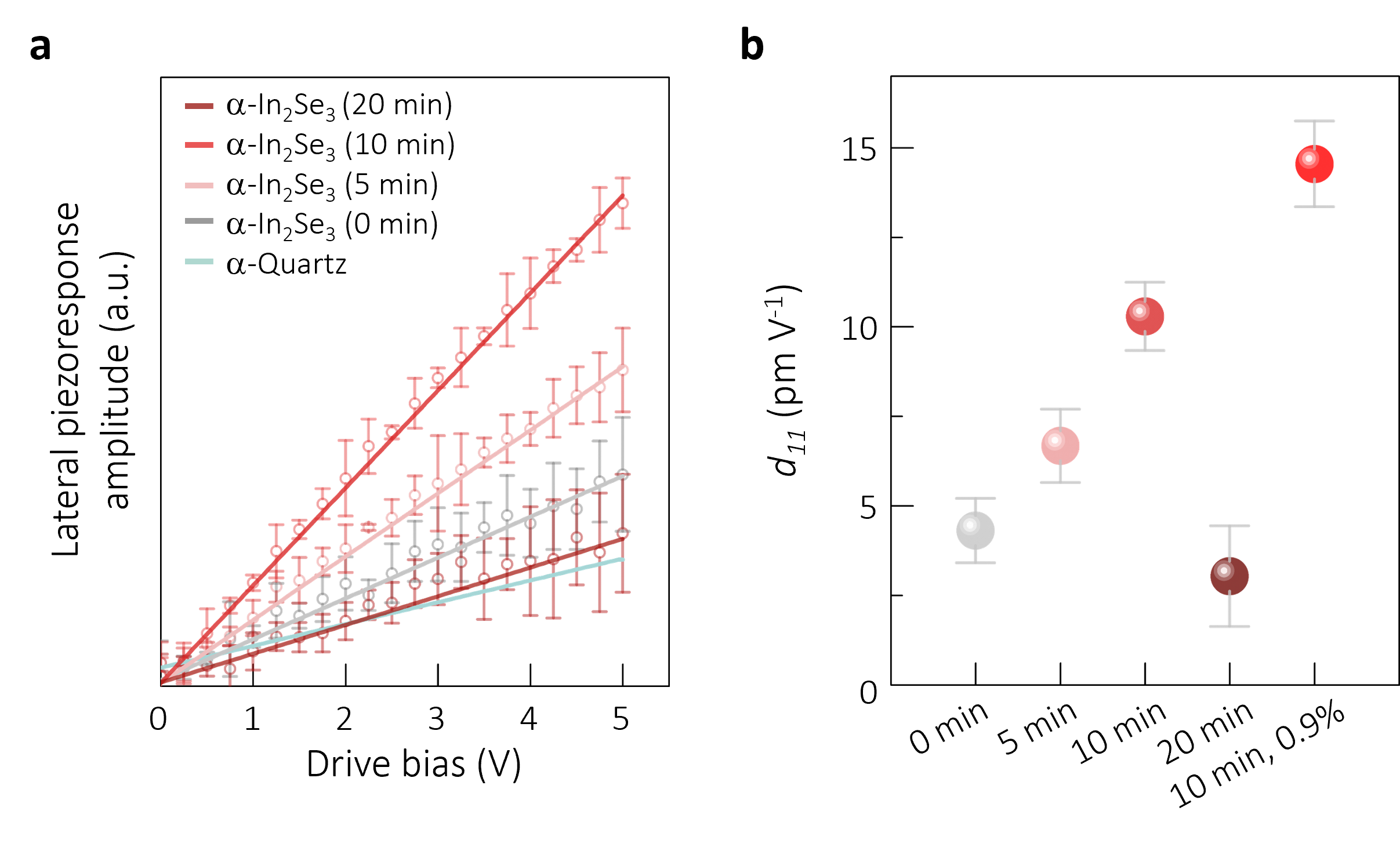


**Figure S9**. (a) Variations in lateral piezoresponse amplitudes with applied bias for α-In_2_Se_3_ nanosheets treated with different plasma exposure time. (b) Resultant *d_11_* values for the different sample conditions.

**Table S1.** Reported piezoelectric energy harvesting performance of the harvesters based on various 2D materials, which was measured under the bending operation, in comparison to our best values.

| **Materials** | **Thickness (nm)** | **Substrate** | **Active area (μm^2^)** | **Bending strain**  **(%)** | **Output voltage**  **(mV)** | **Output current**  **(pA)** | **Power**  **(pW)** | **Power density**  **(μW cm^−2^)** | **Power density**  **(μW cm^−3^)** | **Ref.** |
| --- | --- | --- | --- | --- | --- | --- | --- | --- | --- | --- |
| MoS_2_ | 0.6 | PET | N/A | 0.53 | 15 | 20 | 5.5 × 10^-2^ | 0.2 | 3.3 × 10^6^ | [6] |
| MoS_2_ | 0.7 | PET | 10,000 | 0.48 | 30 | 210 | 0.73 | 7.3 × 10^-3^ | 1.0 × 10^5^ | [12] |
| MoS_2_ | 0.7 | PET | 460* | 0.48 | 20 | 33 | 0.1** | 2.2 × 10^-2^ | 3.1 × 10^5^ | [62] |
| Mo_0.46_W_0.54_S_2_ | 0.9 | PET | 254* | 0.72 | N/A | 250 | 1.8** | 0.7 | 7.8 × 10^6^ | [35] |
| MoSe_2_ | 0.9 | PET | 50* | 0.6 | 60 | 700 | 7.5 | 15 | 1.7 × 10^6^ | [63] |
| WSe_2_ | 0.8 | PET | 5000 | 0.39 | 45 | 100 | 2.54 | 5.1 × 10^-2^ | 6.4 × 10^5^ | [8] |
| WS_2_ | 1 | PI | N/A | 1.56 | 65 | 325 | N/A | 0.6 | 0.6 × 10^7^ | [64] |
| SnS_2_ | 5 | PI | 10^5^ | 0.6 | 42 | 500 | N/A | N/A | N/A | [65] |
| SnS_2_ | 2 – 5 | PI | 36* | 0.6 | 33 | 180 | 2 | 5.6 | ~2.8 × 10^7^ | [66] |
| PbI_2_ | 2.1 | PET | 300* | 0.34 | 29.4 | 20.9 | 0.12 | 4.0 × 10^-2^ | 1.9 × 10^5^ | [67] |
| h-BN | 25 | PI | 0.8* | 0.28 | 50 | 30 | N/A | N/A | N/A | [68] |
| BP | 10 – 20 | PET | 12.5* | 0.56 | 7.8 | 3 | 5.9 × 10^-2^** | 4.7 | 4.7 × 10^6^ | [2] |
| SnS | 1 | Mica | 1000 | 0.7 | 150 | 160 | 24 | 2.4 | 2.4 × 10^7^ | [69] |
| SnS | 5 | PI | 10^5^ | 0.6 | 24 | 230 | N/A | N/A | N/A | [65] |
| SnSe | 60 | PI | 39 | 1.03 | 270 | 297 | 21 | 54 | 9.0 × 10^6^ | [70] |
| γ-InSe | 21 | PET | 100* | 0.31 | 380 | 240 | N/A | N/A | N/A | [71] |
| α-In_2_Se_3_ | 8 | PET | 100* | 0.31 | 363 | 185.4 | N/A | N/A | N/A | [71] |
| α-In_2_Se_3_ | 100 – 200 | PET | N/A | 0.76 | 35.7 | 47.3 | N/A | N/A | N/A | [28] |
| CuInP_2_S_6_ | 90 | PET | N/A | 0.85 | 27.5 | 760 | N/A | N/A | N/A | [72] |
| NbOI_2_ | 70 | PET | 500* | 1.1 | 215 | 180 | N/A | N/A | N/A | [10] |
| α-In_2_Se_3_  (*ε_i_* = 0.9% ) | 90 | PET | 400 | 2.1 | 103.8 | 7.04 × 10^3^ | 300 | 75 | 8.3 × 10^6^ | This work |
| O-doped α-In_2_Se_3_  (*ε_i_* = 0.9% ) | 90 | PET | 400 | 2.1 | 210.4 | 13.5 × 10^3^ | 1690 | ~420 | 4.7 × 10^7^ | This work |

^*^ Dimensions estimated from information provided in each reference.

^**^ Power estimated from information of output voltage under load resistance provided in each reference

(PET: polyethylene terephthalate; PDMS: polydimethylsiloxane; PI: polyimide; PMMA: poly(methyl methacrylate); h-BN: hexagonal boron nitride; BP: black phosphorus)

**Table S2.** Comparison of our best output performance with representative results reported for other piezoelectric thin-film harvesters based on peroovskite oxides and halides, AlN and ZnO.

| **Materials** | **Deposition method** | **Thickness (nm)** | **Substrate** | **Output voltage**  **(V)** | **Output current**  **(μA)** | **Power**  **(μW)** | **Power density**  **(μW cm^−2^)** | **Power density**  **(μW cm^−3^)** | **Mechanical**  **input source (condition)** | **Ref.** |
| --- | --- | --- | --- | --- | --- | --- | --- | --- | --- | --- |
| Pb(Zr_0.53_Ti_0.47_)O_3_ | Sputtering | 2,800 | Stainless steel | 2.6 | N/A | 244 | 308 | 1.1 × 10^6^ | Vibration  (50 Hz) | [73] |
| Pb(Zr,Ti)O_3_ | Sputtering | 3,000 | Stainless steel | 9.4 | N/A | 13.4 | 15.9 | 5.3 × 10^4^ | Pressing  (finger) | [74] |
| Pb(Zr,Ti)O_3_ | Spin-coating | 1,000 | Si | 0.16 | N/A | 2.15 | 0.32 | 3.2 × 10^3^ | Vibration  (462 Hz) | [75] |
| (K_0.45_Na_0.55_)NbO_3_ | Sputtering | 2,200 | Stainless steel | 0.42 | N/A | 1.6 | 0.09 | 4.1 × 10^2^ | Vibration  (393 Hz) | [76] |
| Mn-(K_0.5_Na_0.5_)NbO_3_ | Spin-coating | 1,000 | Si | 0.52 | N/A | 3.6 | 0.18 | 1.8 × 10^3^ | Vibration  (132 Hz) | [77] |
| (Bi_0.5_Na_0.5_)TiO_3_-BaTiO_3_ | Spin-coating | 2,000 | Si | 0.75 | N/A | 2.22 | 9.2 | 4.6 × 10^4^ | Vibration  (42 Hz) | [78] |
| AlN | Sputtering | 500 | Si | 2.48 | N/A | 20.5 | 0.21 | 4.1 × 10^3^ | Vibration  (210 Hz) | [79] |
| AlN | Sputtering | 900 | PI | 0.7 | N/A | 1.4ⅹ10^-3^ | 3.6 × 10^-2^ | 4.0 × 10^2^ | Bending  (N/A) | [80] |
| ZnO | Sputtering | 2,000 | PET | 2.25 | N/A | 0.28 | 0.28 | 1.4 × 10^3^ | Vibration  (370 Hz) | [81] |
| CsPbBr_3_ | Spin-coating | 545 | ITO-PEN | 22.6 | 1.13 | 21.3 | 3.05 | 5.6 × 10^4^ | Bending  (strain, 0.67%) | [29] |
| CsSnI_3_ | Spin-coating | 354 | ITO-PEN | 9.5 | 0.45 | 4.23 | 0.85 | 2.4 × 10^4^ | Bending  (N/A) | [82] |
| MAPbI_3_ | Spin-coating | 486 | ITO-PEN | 23.1 | 1.70 | 182 | 13.1 | 2.7 × 10^5^ | Bending  (strain, 0.47%) | [37] |
| Amorphous CaCu_3_Ti_4_O_12_ | Sputtering | 497 | PEN | 38.7 | 1.24 | 413 | 138 | 2.8 × 10^6^ | Bending (strain, 0.77%) | [42] |
| α-In_2_Se_3_  (*ε_i_* = 0.9% ) | Mechanical exfoliation | 90 | PET | 0.10 | 7.04 × 10^-3^ | 3.0 × 10^-4^ | 75 | 8.3 × 10^6^ | Bending (strain, 2.1%) | This work |
| O-doped α-In_2_Se_3_  (*ε_i_* = 0.9% ) | Mechanical exfoliation | 90 | PET | 0.21 | 1.35 × 10^-2^ | 1.69 × 10^-3^ | ~420 | 4.7 × 10^7^ | Bending (strain, 2.1%) | This work |

(MA: methylammonium; PI: polyimide; PEN: polyethylene naphthalate; PET: polyethylene terephthalate)

**Table S3.** DFT-calculated defect formation energy of all symmetrically inequivalent configurations for each oxygen surface coverage ($\theta_{O}$).

| Oxygen surface coverage ($\boldsymbol{\theta}_{\mathbf{O}}$) | Symmetrically inequivalent configurations | Defect formation energy ($\boldsymbol{\Delta}\boldsymbol{H}^{\mathbf{f}}$) (eV/unit cell) |
| --- | --- | --- |
| 0.16 | config1 | –0.249 |
|  | config2 | –0.236 |
|  | config3 | –0.248 |
| 0.25 | config1 | –0.529 |
|  | config2 | –0.381 |
| 1.00 | config1 | –0.021 |

**Note S2**: Atomic Structure Modeling for the O_2_ Plasma Treatment on In_2_Se_3_

**
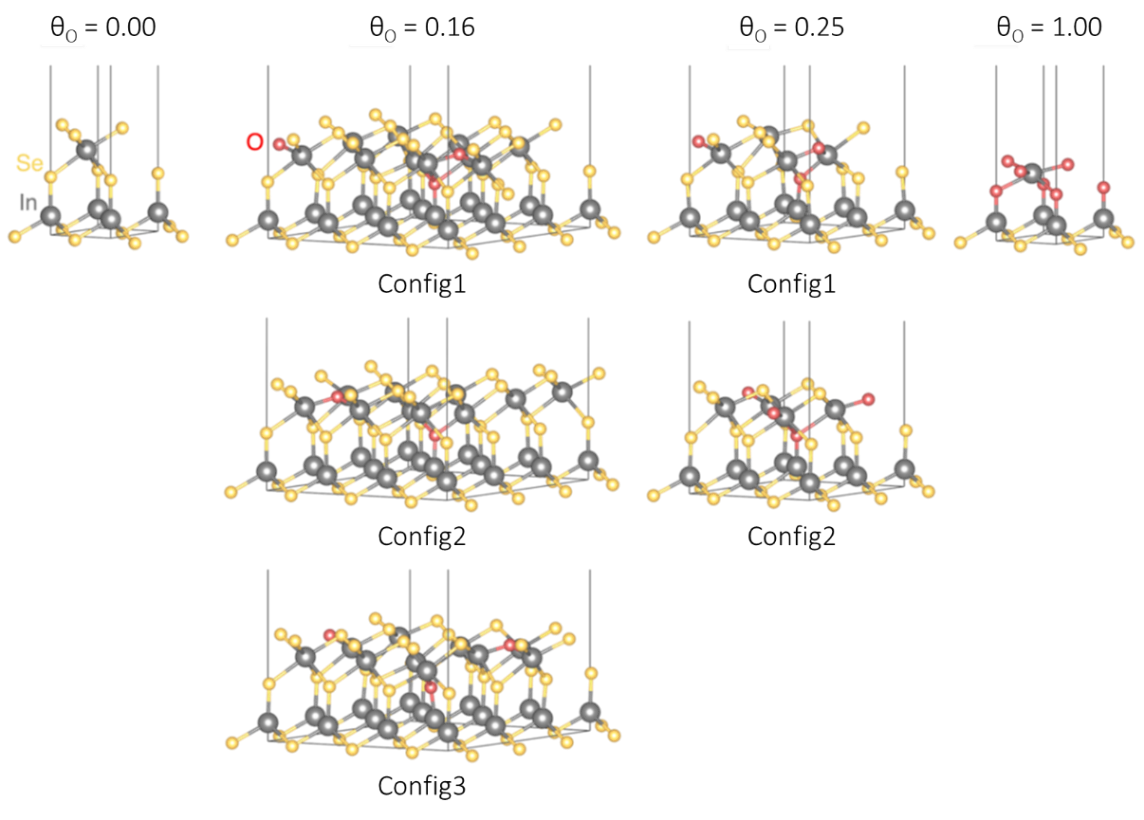
**

**Figure S10.** All symmetrically inequivalent configurations corresponding to the various oxygen surface coverages ($\theta_{O}$). In, Se, and O atoms are represented by gray, yellow, and red spheres, respectively.

All symmetrically inequivalent configurations corresponding to the various surface coverages of oxygen were constructed using the Supercell code (*J. Cheminformatics* **2016**, *8*, 17). There were three symmetrically inequivalent configurations for 0.16$\theta_{O}$, two for 0.25$\theta_{O}$, and one for 1.00$\theta_{O}$, as shown in Figure S10.

To select the configuration with the lowest energy for each oxygen surface coverage, we define the formation energy of the oxygen defect, $\Delta H^{\mathrm{form}}$, as follows:

$\Delta H^{\mathrm{form}}=E_{O-doped \mathrm{In}_{2}\mathrm{Se}_{3}}- E_{\mathrm{In}_{2}\mathrm{Se}_{3}}- \frac{n_{O}}{2}E_{O_{2}}+n_{\mathrm{Se}}E_{\mathrm{Se}}$ (3)

where $E_{O-doped \mathrm{In}_{2}\mathrm{Se}_{3}}$, $E_{\mathrm{In}_{2}\mathrm{Se}_{3}}$, $E_{O_{2}}$, and $E_{\mathrm{Se}}$ are the DFT-calculated ground-state total energies for the oxygen-containing In_2_Se_3_, defect-free In_2_Se_3_, molecular O_2_, and bulk Se, respectively. Here, *n*_o_ and *n*_Se_ are the number of O and Se atoms, respectively. The DFT-calculated defect formation energies are listed in Table S3.
